# Common genetic variation in and near the melanocortin 4 receptor gene (*MC4R*) is associated with body mass index in American Indian adults and children

**DOI:** 10.1007/s00439-014-1477-6

**Published:** 2014-08-08

**Authors:** Yunhua L. Muller, Marie S. Thearle, Paolo Piaggi, Robert L. Hanson, Duncan Hoffman, Brittany Gene, Darin Mahkee, Ke Huang, Sayuko Kobes, Susanne Votruba, William C. Knowler, Clifton Bogardus, Leslie J. Baier

**Affiliations:** Phoenix Epidemiology and Clinical Research Branch, National Institute of Diabetes and Digestive and Kidney Disease, National Institutes of Health, 445 North 5th street, Phoenix, AZ 85004 USA

## Abstract

Six rare functional coding mutations were previously identified in melanocortin 4 receptor (*MC4R*) in 6,760 American Indians. Individuals heterozygous for one of these mutations become obese while young. We now investigate whether common non-coding variation near *MC4R* also contributes to obesity. Fifty-six tag single-nucleotide polymorphisms (SNPs) were genotyped in 3,229 full-heritage Pima Indians, and nine of these SNPs which showed evidence for association were genotyped in additional 3,852 mixed-heritage American Indians. Associations of SNPs with maximum body mass index (BMI) in adulthood (*n* = 5,918), BMI *z* score in childhood (*n* = 5,350), percent body fat (*n* = 864), energy expenditure (*n* = 358) and ad libitum food intake (*n* = 178) were assessed. Conditional analyses demonstrated that SNPs, rs74861148 and rs483125, were independently associated with BMI in adulthood (*β* = 0.68 kg/m^2^ per risk allele, *p* = 5 × 10^−5^; *β* = 0.58 kg/m^2^, *p* = 0.002, respectively) and BMI *z* score in childhood (*β* = 0.05, *p* = 0.02; *β* = 0.07, *p* = 0.01, respectively). One haplotype (frequency = 0.35) of the G allele at rs74861148 and the A allele at rs483125 provided the strongest evidence for association with adult BMI (*β* = 0.89 kg/m^2^, *p* = 5.5 × 10^−7^), and was also associated with childhood BMI *z* score (*β* = 0.08, *p* = 0.001). In addition, a promoter SNP rs11872992 was nominally associated with adult BMI (*β* = 0.61 kg/m^2^, *p* = 0.05) and childhood BMI *z* score (*β* = 0.11, *p* = 0.01), where the risk allele also modestly decreased transcription in vitro by 12 % (*p* = 0.005). This risk allele was further associated with increased percent body fat (*β* = 2.2 %, *p* = 0.002), increased food intake (*β* = 676 kcal/day, *p* = 0.007) and decreased energy expenditure (*β* = −53.4 kcal/day, *p* = 0.054). Common and rare variation in *MC4R* contributes to obesity in American Indians.

**Electronic supplementary material:**

The online version of this article (doi:10.1007/s00439-014-1477-6) contains supplementary material, which is available to authorized users.

## Introduction

The melanocortin pathway in the human hypothalamus plays a pivotal role in regulating food intake and energy homeostasis. Melanocortin 4 receptor (*MC4R*) haploinsufficiency is the most common monogenic cause of obesity, with a prevalence of 1.7–3.0 % in obese individuals (Calton et al. 2009; Hainerová et al. 2007; Hinney et al. 2006). We previously identified 10 rare non-synonymous mutations by sequencing the entire coding region of the *MC4R* gene in 6,760 American Indians, predominately of Pima Indian heritage. In vitro luciferase assays showed that six of these mutations had a functional impact on the receptor’s activity. The population prevalence of these combined functional coding mutations in *MC4R* was 2.4 %, and in these individuals the rate of increase in body mass index (BMI) and risk of type 2 diabetes (T2D) was more apparent during childhood as compared with adulthood (Thearle et al. 2012). Since obesity is very common among American Indians, the high prevalence of this disease cannot be solely explained by these rare *MC4R* mutations. Genome-wide association studies (GWASs) in Caucasians and other ethnic groups have identified common non-coding variants near *MC4R* that are reproducibly associated with fat mass, body weight and risk of obesity (Loos et al. 2008; Willer et al. 2009; Speliotes et al. 2010). Several risk alleles in *MC4R*, which are common and consistently associated with obesity/BMI in studies of Caucasians (e.g., rs17782313, rs17700633 and rs571312), are uncommon in American Indians (frequency = 0.03, 0.005 and 0.003, respectively) despite American Indians having much higher rates of obesity than Caucasians. This raises the possibility that different variants in *MC4R* may contribute to obesity in American Indians. Therefore, in the current study, we investigate the relative contribution of common variation across *MC4R* on risk of obesity in American Indians, and further explore the effect of common *MC4R* variation on energy intake and energy expenditure measures.

## Methods

### Subjects

Subjects are participants of a longitudinal study of the etiology of T2D among the Gila River Indian Community in Arizona, where most of the residents are Pima Indians or Tohono O’odham (a closely related tribe) (Knowler et al. 1990). Individuals participated in biennial examinations that included measurements of BMI, and fasting and 2-h glucose and insulin concentrations during a 75-g oral glucose tolerance test (OGTT). Diabetes was determined by prior clinical diagnosis or an oral glucose tolerance test according to the criteria of American Diabetes Association (The Expert Committee on the Diagnosis and Classification of Diabetes Mellitus 1997). For analysis of maximum BMI during adulthood, the highest BMI after the age of 15 years recorded from a non-diabetic exam was considered (*n* = 5,918, males 44 %, BMI 35.1 ± 8.4 kg/m^2^, age 29.7 ± 11.4 years). For analysis of childhood obesity, the maximum sex- and age-specific BMI *z* score (Pima based) was identified between the ages of 5 and 20 years (*n* = 5,350, males 45 %, BMI 27.2 ± 6.7 kg/m^2^, age 13.8 ± 3.9 years). Data including BMI measured at multiple non-diabetic exams (range 1–17 exams for a given individual during adulthood; 1–8 exams during childhood) between the years 1965 and 2004 were also analyzed. The mean BMI from all non-diabetic exams during adulthood was 32.7 ± 8.0 kg/m^2^, and the mean BMI during childhood was 25.5 ± 6.2 kg/m^2^ (*z* scores were converted to BMI units using the mean and standard deviation of 12-year-old Pima females). Among these subjects, 3,229 were full-heritage Pima Indian; the remaining 3,852 subjects were mixed heritage, on average one-half Pima and three-quarters American Indian. DNA from all of these American Indians had been previously sequenced for *MC4R* coding mutations, and the 167 individuals had a rare functional coding mutation. With the severe functional impairment caused by the rare coding variants in these individuals (BMI 39.0 ± 7.6 kg/m^2^ in adulthood, 32.7 ± 8.0 kg/m^2^ in childhood) (Thearle et al. 2012), the additional influence of any, presumably more moderate, functional effects of common variants is likely negligible. Therefore, these 167 individuals were excluded from the present study.

### Quantitative metabolic traits and ad libitum food intake study

Among the subjects who were genotyped, 538 non-diabetic American Indians (predominately full-heritage Pima Indians, males 58 %, age 27 ± 6 years, BMI 34 ± 8 kg/m^2^) had undergone detailed metabolic studies in our Clinical Research Unit (CRU). Body composition was estimated by underwater weighing until 1994 and by dual energy X-ray absorptiometry (DPX-1, Lunar Radiation Corp.) thereafter (Tataranni and Ravussin 1995). Energy expenditure and sleeping metabolic rate over 24 h were assessed in 358 non-diabetic subjects using whole-room indirect calorimetry as previously described (Ravussin et al. 1986). Spontaneous physical activity (SPA) was detected by radar sensors and expressed as percentage of time in motion over 24 h. Sleeping metabolic rate was defined as the average energy expenditure between 01:00 am and 05:00 am during which SPA was <1.5 % (Piaggi et al. 2013a) and the thermic effect of the last meal was undetected (Piaggi et al. 2013b). As a confirmatory measure, resting metabolic rate was assessed by indirect calorimetry using a ventilated hood system before the hyperinsulinemic, euglycemic clamp as previously described (Lillioja et al. 1993; Bogardus et al. 1986).

Also genotyped were a subset of adults who had participated in an ad libitum food intake study as previously described (Pannacciulli et al. 2007). Among 178 participants who had both genotype and food intake data (males 60 %, age 34.7 ± 9.0 years, BMI 32.7 ± 8.0 kg/m^2^), 34 % were full-heritage Pima Indians, 41 % were mixed-heritage American Indians, and 25 % were Caucasians. Food intake was also assessed in a separate study of the relationship between in utero exposures and maternal environmental influences on food intake in full-heritage Pima Indian children. Fifty-five of them had genotype and food intake data (males 49 %, age 9.5 ± 1.2 years, BMI 25.4 ± 6.7 kg/m^2^) (Gluck et al. 2009). Ad libitum food intake in both studies was assessed over 3 days by an automated vending machine system in our CRU (Pannacciulli et al. 2007).

### Selection and genotyping of *MC4R* tag SNPs

Common variation (minor allele frequency, mAF ≥ 0.01) across a ~510 kb region encompassing *MC4R* (chr18:57778138-58288450, ~250 kb flanking each side of the gene, GRCh37/hg19) was identified from whole-genome sequence data (30–40× coverage) of 234 Pima Indians (Complete Genomics Inc., Mountain View, CA; Illumina, San Diego, CA, USA) (Muller et al. 2014). Linkage Disequilibrium (LD) between variants was determined using Haploview (version 4.2). Tag SNPs were selected using the Tagger algorithm with a pair-wise *r* ^2^ ≥ 0.8. SNPs were genotyped using the TaqMan Open Array System (Applied Biosystems, Carlsbad, CA, USA) or BeadXpress System (Illumina, San Diego, CA, USA).

### Statistical analyses

Statistical analyses were performed using the software of the SAS Institute (Cary, NC, USA). The association of quantitative traits including maximum BMI, maximum childhood BMI *z* score and metabolic traits with genotypes was analyzed by linear regression using the generalized estimating equation procedure (GEE) to account for family membership (sibship). *p* values were adjusted for multiple covariates as indicated. The individual estimate of the proportion of European ancestry was derived by the method of Hanis et al. (1986) from 45 informative markers with large differences in allele frequency between populations (Tian et al. 2007) for use as a covariate in all analyses. The association between genotype and adult BMI or childhood *z* score was also analyzed using all longitudinal non-diabetic BMI measurements from an individual. A linear mixed model (PROC MIXED) was fitted that included genotype as a fixed effect along with age, sex, birth year and heritage as covariates. The model also included random effects representing sibship and individual (to account for multiple examinations within an individual). To model the relationship between multiple examinations within an individual, an autoregressive correlation structure was used as previously described (Ma et al. 2010). The difference in change in BMI over time between genotypes was assessed with mixed models to account for repeated measures using a compound symmetry covariance structure and modeling BMI change over time as a linear function for simplicity. The general association of genotype with T2D was assessed with logistic regression analysis and adjusted for covariates, which included age, sex, birth year and heritage. The model was fitted with GEE to account for correlation among siblings.

Conditional analyses were also conducted in which a SNP of interest was added to a model containing one or more additional SNPs. Haplotypes were determined by a modification of the zero recombinant haplotyping method as previously described (Vozarova de Courten et al. 2005). The MLINK program is used to calculate the probability that each individual carries one or two copies of each haplotype, given their genotypes and the genotypes of their family members. These probabilities were then analyzed in regression models in a fashion analogous to individual SNPs.

### Functional analysis of the promoter activity of the *MC4R* SNP

A 634-bp DNA fragment of the *MC4R* promoter region containing either an A or G nucleotide at position chr18:58040587 for rs11872992 was amplified with primers forward 5′-ATCTCGAGTGTTAGGGGCTGTA-3′ and reverse 5′-TCAGGTACCAATAGAGAAATATGG-3′ (*Xho*I and *Kpn*I restriction sites are underlined, respectively). PCR products were inserted at *Kpn*I and *Xho*I sites upstream of the pGL3-Basic luciferase reporter vector (Promega, Madison, WI, USA).

Transient transfection in the murine N-42 hypothalamus cell line (Cellutions Biosystems, Inc., Burlington, ON, Canada) and dual luciferase assays (Promega, Madison, WI, USA) were performed using the modified method as previously described (Thearle et al. 2012; Muller et al. 2003). Four separate transfections were performed and each transfection was repeated in triplicate. The statistical difference in mean luciferase activity between the G and A allele at rs11872992 was analyzed by an unpaired *t* test.

## Results

### Association of *MC4R* tag SNPs with adult BMI and childhood BMI *z* score

SNPs (*n* = 56) tagging common variation (mAF ≥ 0.01) in Pima Indians across a ~510 kb region encompassing *MC4R* (Supplemental Figure) were initially genotyped in full-heritage Pima subjects from the Gila River Indian Community (*n* = 3,229, Supplementary Table 1). SNPs that showed a trend for association (7 SNPs with *p* ≤ 0.01) with either maximum BMI in adulthood or maximum BMI *z* score in childhood, or were predicted to be damaging using the Ingenuity Variant Analysis (rs11872992) were further genotyped in all remaining subjects (*n* = 3,852, mixed-heritage American Indians, Supplementary Table 1). In addition, rs17782313 which is the highly replicated common SNP near *MC4R* in other populations (Loos et al. 2008; Willer et al. 2009; Speliotes et al. 2010) was also genotyped in all study subjects. These nine SNPs were analyzed for associations with maximum BMI in adulthood (*n* = 5,918) and maximum BMI *z* score in childhood (*n* = 5,350) in all American Indians as shown in Table 1. A mixed model analysis was also performed using longitudinal data for BMI at multiple exams for each individual, and statistical results were comparable with association data for maximum BMI (Table 1). For further comparison, association data for BMI from the large GIANT (Genetic Investigation of ANthropometric Traits) meta-analysis of Caucasians (Speliotes et al. 2010) and a case–control study of the Belgian population (Beckers et al. 2011) are also shown in Table 1.

**Table 1:** Associations of 8 tag SNPs and rs17782313 in the *MC4R* region with adult BMI and childhood BMI *z* score in all American Indians

SNPs	All American Indians (adult, *n* = 5,918; children, *n* = 5,350)										Caucasian (GIANT)^a^ (*n* = 123,795)			Caucasian (Belgian)^b^ (*n* = 1,361)		
	Allele R/N	RAF	Age	R/R	R/N	N/N	Maximum BMI		Multiple BMI		Allele R/N	RAF	*p* value	AlleleR/N	RAF	*p* value
							*β*	*p* value	*β*	*p* value						
rs74861148	G/A	0.38	Adult BMI	36.9	35.3	34.1	0.675	**5** **×** **10** ^**−5**^	0.538	**0.0007**	N/A			N/A		
Downstream			BMI *z* score	0.41	0.31	0.25	0.053	**0.018**	0.055	**0.005**						
rs8087522^c^	A/G	0.45	Adult BMI	35.8	35.2	34.1	0.562	**0.0003**	0.517	**0.0007**	A/G	0.38	0.472	A/G	0.32	0.54
Promoter			BMI *z* score	0.40	0.32	0.23	0.075	**0.0007**	0.069	**0.0004**						
rs11872992^c^	G/A	0.93	Adult BMI	35.1	34.2	32.4	0.613	**0.049**	0.545	0.057	G/A	0.87	0.722	G/A	0.88	**0.03**
Promoter			BMI *z* score	0.33	0.18	0.01	0.105	**0.011**	0.083	**0.028**						
rs62097832	G/A	0.53	Adult BMI	36.2	35.1	33.9	0.613	**0.0001**	0.559	**0.0003**	N/A			N/A		
Upstream			BMI *z* score	0.42	0.28	0.21	0.083	**0.0002**	0.082	**3** **×** **10** ^**−5**^						
rs11661166	G/A	0.56	Adult BMI	35.8	35.0	34.2	0.485	**0.002**	0.460	**0.003**	G/A	0.46	0.306	N/A		
Upstream			BMI *z* score	0.40	0.29	0.21	0.081	**0.0003**	0.071	**0.0003**						
rs8088123	C/A	0.88	Adult BMI	35.1	34.7	35.7	0.390	0.097	0.197	0.381	C/A	0.93	0.064	N/A		
Upstream			BMI *z* score	0.33	0.20	0.34	0.085	**0.009**	0.086	**0.003**						
rs483145	A/T	0.80	Adult BMI	35.5	34.3	33.9	0.583	**0.002**	0.357	0.052	A/T	0.62	0.323	N/A		
Upstream			BMI *z* score	0.35	0.23	0.23	0.065	**0.010**	0.055	**0.018**						
rs474421	C/T	0.55	Adult BMI	35.3	35.2	34.9	0.137	0.372	0.197	0.181	C/T	0.53	0.115	N/A		
Upstream			BMI *z* score	0.32	0.32	0.26	0.028	0.204	0.044	**0.022**						
rs17782313^d^	C/T	0.03	Adult BMI	26.1	32.9	35.2	−0.26	0.613	-0.37	0.440	C/T	0.28	**8** **×** **10** ^**−22**^	C/T	0.26	**0.002**
Upstream			BMI *z* score	0.55	0.25	0.31	0.073	0.276	0.042	0.493						

The maximum adult BMI (kg/m^2^) was recorded from a non-diabetic exam at the age ≥15 years. The maximum childhood BMI is the age- and sex-specific *z* score from an exam at the ages of 5–20 years. BMI is given as an unadjusted mean calculated from the maximum value for each person. A mixed model analysis was also performed using longitudinal data for BMI at multiple non-diabetic exams for each individual. For adult BMI analyses (maximum or multiple), BMI was log_e_ transformed, and the regression coefficient (*β*) was exponentiated to obtain the effect estimate for each risk allele, expressed as a multiplier. For presentation in the table, a multiplier was converted to the effect size in kg/m^2^ based on a percentage of risk increase or decrease in mean population BMI (i.e., a multiplier of 1.02 is equivalent to 2 % × 35.12 kg/m^2^ = 0.70 kg/m^2^). Beta for childhood BMI (maximum or multiple) was expressed as *z* score per copy of the risk allele. *β* and *p* values were adjusted for age, sex, birth year and heritage. Bold values indicate *p* ≤ 0.05. The risk allele (given first) for rs17782313 is defined as the observed risk allele in European studies, while for other SNPs it is defined as the allele with a higher BMI in Pima Indians. SNPs are listed in an order corresponding to the chromosome position

*R* risk allele, *N* non-risk allele, *RAF* risk allele frequency

^a^GIANT meta-analysis (Speliotes et al. 2010)

^b^A case–control study of the Belgian population (Beckers et al. 2011)

^c^SNP is predicted to cause a loss of promoter function by the Ingenuity Variant Analysis

^d^Indicates the established obesity variant in European populations

The strongest single SNP association with maximum BMI in American Indian adults was observed for rs74861148 with a risk allele frequency (RAF) of 0.38, mapping ~220 kb downstream of *MC4R*. Rs74861148 was associated with maximum BMI in 5,918 adults [*β* = 0.68 (95 % CI 0.35, 1.01) kg/m^2^ per risk allele, *p* = 5 × 10^−5^, adjusted for age, sex, birth year and heritage]. Individuals who were homozygous for the risk allele (GG) had an increased adjusted BMI of 1.4 kg/m^2^ compared with those with the non-risk allele (AA). This SNP was also nominally associated with maximum BMI *z* score in 5,350 children [*β* = 0.05 (0.01, 0.10), adjusted *p* = 0.02]. In Caucasian and African populations, this SNP is monomorphic for the G (risk) allele.

Single marker analyses identified weaker evidence for association between maximum BMI in adulthood and six other tag SNPs: rs8087522, rs11872992, rs62097832, rs11661166, rs8088123 and rs483145 (adjusted *p* ≤ 0.05, Table 1). All tag SNPs showing any evidence for association were in high *D*’ (0.52–1.00), although in low *r* ^2^ (0.01–0.67). To determine the extent to which rs75861148 contributed to each of these other associations, conditional analyses were conducted in a step-wise fashion. Controlled for rs74861148 genotypes, only rs483145 (mapping ~128 kb upstream of *MC4R* with RAF = 0.80) remained associated with maximum BMI (conditional *p* = 0.02). When both rs74861148 and rs483145 were included in the model, none of other SNPs remained associated with BMI (all conditional *p* > 0.05), suggesting that both rs74861148 and rs483145 (*D*’ = 0.58, *r* ^2^ = 0.06) independently contribute to the BMI associations in American Indians. However, rs483145 was not associated with BMI in the GIANT meta-analysis (*p* = 0.32; Table 1).

**Fig. 1:**
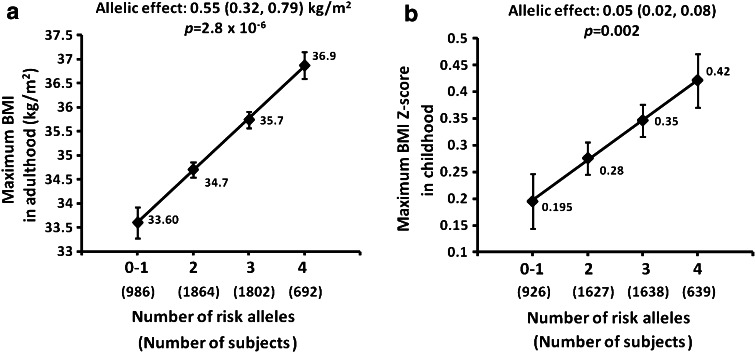
Additive effects on maximum BMI in American Indian adults (**a**) and maximum BMI *z* score in children (**b**) with increasing numbers of risk alleles of rs74861148 and rs483154. Only 3.3 % subjects carried non-risk alleles at both loci (#risk allele = 0), thus these subjects were combined with those who carried one risk allele from either locus (#risk allele = 1). Maximum BMI values (mean ± SE), allelic effects (95 % CI) and *p* values were adjusted for age, sex, birth year and heritage

To quantify the additive effects of rs74861148 and rs483145 on maximum BMI in American Indians, we analyzed BMI of individuals according to the number of risk alleles at these SNPs. Each additional risk allele increased BMI by 0.55 (0.32, 0.79) kg/m^2^ in adulthood (Fig. 1a, *p* for trend = 2.8 × 10^−6^, adjusted for age, sex, birth year and heritage). Among 5,918 subjects, only 3.3 % were homozygous for non-risk alleles at both loci, and there was no difference in BMI (*p* > 0.05) between this group and those who carry one of the four risk alleles, hence these two groups were combined (#risk allele = 0–1). Individuals with four risk alleles (12.9 % of the population) had the highest adjusted BMI compared with those with 0 or 1 risk allele (18.5 % of the population, adjusted ΔBMI = 3.3 kg/m^2^). In children, each risk allele increased BMI *z* score by 0.05 (0.02, 0.08), corresponding to a BMI increase of 0.30 (0.12, 0.49) kg/m^2^ (Fig. 1b, adjusted *p* for trend = 0.002). The adjusted Δ*z* score between individuals with 0–1 risk alleles and those with four risk alleles was 0.23, corresponding to ΔBMI of 1.4 kg/m^2^. Together, the common genotypes of rs74861148 and rs483145 accounted for 0.50 and 0.26 % of the variance in liability for adulthood and childhood BMI, respectively.

### Haplotype effects of *MC4R* SNPs on maximum BMI and the rate of BMI change

**Fig. 2:**
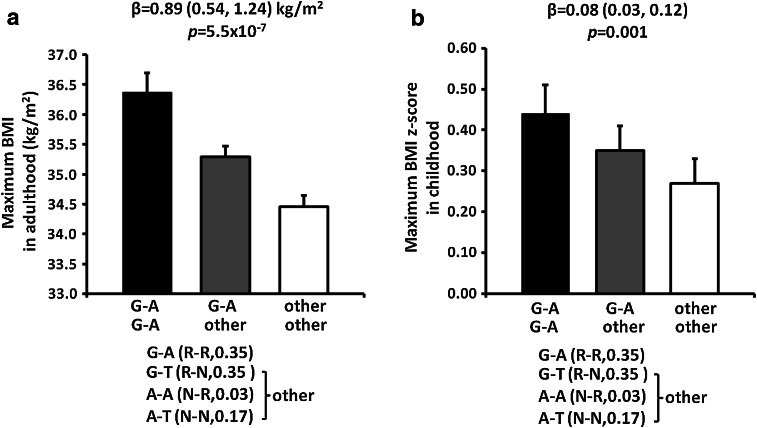
Haplotype effects of rs74861148 and rs483154 on maximum BMI in American Indian adults (**a**) and maximum BMI *z* score in children (**b**). Haplotype frequency is indicated in the parenthesis. Adult BMI and BMI *z* score are presented as an adjusted mean ± SE for each haplotype group. *β* represents the effect per copy of the risk haplotype. *p* values (additive model) and *β* (95 % CI) were adjusted for age, sex, birth year and heritage

**Fig. 3:**
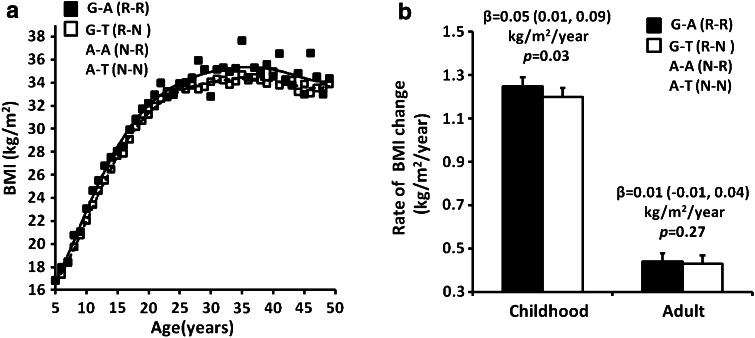
**a** Haplotype effects of rs74861148 and rs483154 on the lifetime BMI trajectory (age 5–50 years). **b** Haplotype effects of rs74861148 and rs483154 on the rate of BMI change in American Indian children and adults. The risk haplotype carriers (at least one copy) were compared with the non-risk haplotype carriers (no copies). BMI is given as an unadjusted mean at each age group. Rates of BMI change (mean ± SE), beta (95 % CI) and *p* values were adjusted for sex, birth year and heritage

Haplotype analyses for these two independent SNPs rs74861148 (G/A) and rs483145 (A/T) showed that three out of four haplotypes were common in American Indians (G–A, frequency = 0.35; G–T, frequency = 0.03; A–A, frequency = 0.44; A–T, frequency = 0.17). There were no significant differences in maximum BMI between individuals carrying haplotypes G–T, A–A or A–T. However, individuals with the haplotype G–A (G risk allele at rs74861148 and A risk allele at rs483145) had a higher maximum BMI as compared with other three haplotypes combined (G–T, A–A and A–T) [Fig. 2a, *β* = 0.89 (0.54, 1.24) kg/m^2^ per copy of the G–A haplotype, *p* = 5.5 × 10^−7^, adjusted for age, sex, birth year and heritage]. In children, those carrying the G–A haplotype also had a higher maximum BMI *z* score as compared with those carrying all other haplotypes combined [Fig. 2b, *β* = 0.08 (0.03, 0.12) per copy of the G–A haplotype, corresponding to BMI of 0.49 (0.12, 0.73) kg/m^2^, adjusted *p* = 0.001]. BMI change over time between the ages of 5–50 years was evaluated in 4,505 American Indians children (13,168 total visits, 3.0 ± 1.5 visits per subject) and 4,160 adults (12,582 total visits, 3.0 ± 2.3 visits per subject) with longitudinal BMI measures before developing diabetes. The lifetime BMI trajectory of the risk haplotype (carriers, at least one copy) vs. non-risk haplotype (non-carriers, no copies) is shown in Fig. 3a. Subjects with the risk haplotype G–A had a consistently higher BMI than subjects with the non-risk haplotype at almost every age. In childhood, the rate of BMI change was 1.25 ± 0.04 kg/m^2^/year in the risk haplotype carriers compared with 1.20 ± 0.04 kg/m^2^/year in the non-risk haplotype carriers [Fig. 3b, *β* = 0.05 (0.01, 0.09) kg/m^2^/year, *p* = 0.03, adjusted for sex, birth year and heritage, *n* = 4,505]. In adulthood, the slope of BMI change stabilized and was no longer greater in the carriers than that in the non-carriers [Fig. 3b, *β* = 0.01 (−0.01, 0.04) kg/m^2^/year, adjusted *p* = 0.27, *n* = 4,160].

### Replication of *MC4R* SNPs in other studies

The strongest signal for BMI near *MC4R* in the GIANT meta-analysis is rs17782313 with a risk allele frequency of *C* = 0.28 (Table 1). The C allele is much less common in American Indians (AF = 0.03) and was not associated with adult BMI or childhood *z* score (Table 1). Conversely, the tag SNP rs74861148 associated with BMI in American Indians was not informative (monomorphic) in Caucasians. However, a smaller study of *MC4R* in a Belgian case/control study (1,049 obese/312 control) reported that a promoter SNP rs11872992 was associated with obesity (*p* = 0.03, Table 1), and this signal was independent from rs17782313 (Beckers et al. 2011). In our study, this promoter SNP rs11872992 (RAF = 0.93) also had a nominal association with maximum BMI in adulthood [Table 1, *β* = 0.61 (0.01, 1.23) kg/m^2^, *p* = 0.05, *n* = 5,918) and maximum BMI *z* score in childhood [β = 0.11 (0.02, 0.19), *p* = 0.01, *n* = 5,350], where the risk allele was consistent with the Belgian study. This variant is located 586 bp from the translational start site of *MC4R*, and predicted to cause a loss of promoter function by the Ingenuity Variant Analysis (www.ingenuity.com) by disrupting a putative transcription factor binding site for GATA2. Therefore, although our conditional analyses indicated that the weaker association of rs11872992 with BMI in American Indians could largely be explained by the stronger association of rs74861148 (*D*’ = 0.69), we pursued additional studies with this variant.

### In vitro functional analyses of promoter activity of rs11872992

**Fig. 4:**
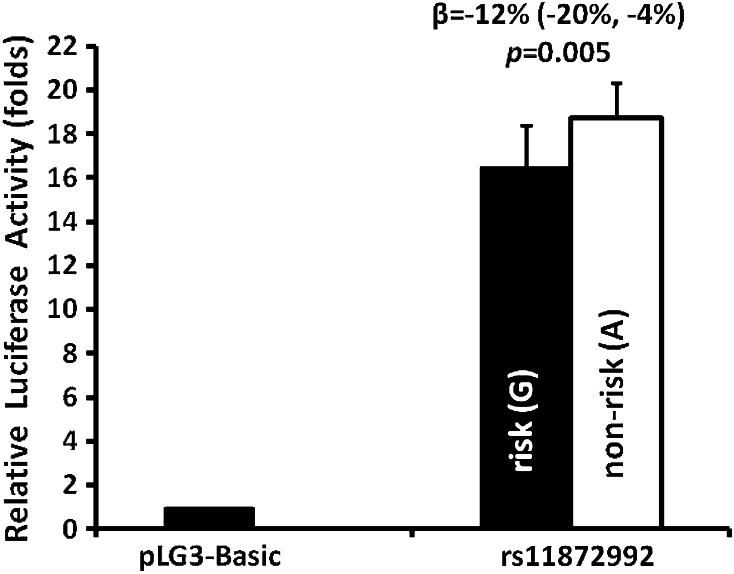
In vitro functional analyses of promoter activity of rs11872992. Relative luciferase activity (fold change) was expressed as a ratio of firefly luciferase activity to renilla luciferase activity, and further normalized to pGL3-basic luciferase activity. Data are presented as mean ± SD, *n* = 12 transfections. The statistical difference in the averaged activity was analyzed by an unpaired *t* test

The promoter activity of rs11872992 was assessed by an in vitro luciferase assay in a murine hypothalamus cell line (Fig. 4). Overall, the *MC4R* promoter region containing rs11872992 increased luciferase activity by ~18-fold, as compared with a promoter-less luciferase vector (pGL3-Basic), indicating the strong promoter activity in this region. The DNA construct with the BMI risk allele G at rs11872992 had a modest, but consistent decrease [*β* = −12 % (−20, −4 %) in promoter activity in contrast to the non-risk A allele (*p* = 0.005). SNP rs8087522 was also predicted to cause a loss of promoter function (Ingenuity Variant Analysis); however, our in vitro functional analyses did not detect a difference between the alleles of rs8087522 (data not shown).

### Additional phenotype analyses of the functional promoter SNP rs11872992

To assess the effect of rs11872992 on longitudinal changes in BMI, we performed mixed model analyses to include data from all available exams and analyzed differences in change in BMI or childhood BMI *z* score over time. Rs11872292 had a low allele frequency for the A allele (mAF = 0.07), therefore the heterozygous GA and homozygous AA genotypes were combined for analyses. During childhood, the rate of BMI change was 1.27 kg/m^2^/year in subjects with the risk GG alleles vs. 1.20 kg/m^2^/year in subjects with the non-risk GA + AA alleles [*β* = 0.07 (0.02, 0.11) kg/m^2^/year, adjusted *p* = 0.004]. In adulthood, the accelerated rate of BMI gain continued in individuals with the risk alleles GG compared with those with the non-risk alleles GA + AA [0.26 vs. 0.22 kg/m^2^/year, *β* = 0.04 (0.01, 0.07) kg/m^2^/year, adjusted *p* = 0.01]. Many of these subjects (*n* = 7,667) also had data available for T2D status, and rs11872992 was associated with increased risk for T2D such that individuals with the risk allele for BMI (G allele) had a higher prevalence of T2D with an odds ratio of 1.23 per copy of the risk allele G (95 % CI 1.03, 1.46, *p* = 0.02, adjusted for age, sex, birth year and heritage). However, the association with T2D was attenuated after adjustment for maximum BMI (adjusted *p* = 0.09), suggesting that the finding was mainly attributable to increased BMI.

A subset of the individuals (*n* = 864) with genotypic data also had detailed measures of metabolic traits related to obesity (Table 2). The risk allele G for BMI at rs11872992 was associated with higher percentage of body fat [Table 2, *β* = 2.2 (0.8, 3.6)  %, *p* = 0.002, adjusted for age, sex and heritage], higher fat mass [(*β* = 3.5 (0.9, 6.1) kg, adjusted *p* = 0.008)], higher waist [*β* = 1.4 (0.01, 2.8) inch, adjusted *p* = 0.05)] and thigh circumferences [*β* = 0.7 (0.02, 1.4) inch, adjusted *p* = 0.04], but not associated with the ratio of waist to thigh (*p* = 0.58) and fat-free mass (*p* = 0.15). In whole-room indirect calorimetry study (*n* = 358), the risk allele at rs11872992 was also nominally associated with a lower 24-h energy expenditure [*β* = −53.4 (−107.9, 1.1) kcal/day, *p* = 0.054, adjusted for age, sex, fat mass, fat-free mass, SPA and heritage]. However, there were no differences in sleeping metabolic rate (*p* = 0.23, *n* = 358) and fasting resting metabolic rate (*p* = 0.80, *n* = 519, measured by a ventilated hood indirect calorimetry).

**Table 2:** Association of rs11872992 with metabolic traits and ad libitum energy intake in non-diabetic American Indians

	GG risk/risk	rs11872992 (G/A) (mean ± SD)		
		GA + AA risk/non	*β* (95 % CI)	*p* value
Subjects, body composition (*n*)	760	104		
Percentage of body fat	33.8 ± 8.4	31.0 ± 8.7	2.2 (0.8, 3.6)	**0.002**
Fat mass (kg)	33.4 ± 14.5	29.3 ± 13.9	3.5 (0.9, 6.1)	**0.008**
Fat-free mass (kg)	62.5 ± 14.4	61.4 ± 14.0	1.8 (−0.6, 4.1)	0.145
Waist (inch)	43.1 ± 7.0	41.5 ± 7.4	1.4 (0.01, 2.84)	**0.048**
Thigh (inch)	25.9 ± 3.4	25.1 ± 3.6	0.7 (0.02, 1.44)	**0.044**
Waist/thigh	1.67 ± 0.18	1.65 ± 0.20	0.01 (−0.03, 0.05)	0.584
Subjects, hyperinsulinemic, euglycemic clamp (*n*)	452	67		
Resting metabolic rate (kcal/day)	1806 ± 338	1,748 ± 304	7.5 (−50.1, 65.1)	0.799
Subjects, whole-room indirect calorimetry (*n*)	313	45		
24-h energy expenditure (kcal/day)	2,371 ± 407	2,324 ± 365	−53.4 (−107.9, 1.1)	0.054
Sleeping metabolic rate (kcal/day)	1,678 ± 284	1,616 ± 244	−23.2 (−61.6, 14.6)	0.229
Subjects, ad libitum energy intake (*n*)	147	31		
Carbohydrate intake (g/day)	553 ± 174	472 ± 139	81.5 (21.4, 141.6)	**0.009**
Fat intake (g/day)	191 ± 68	159 ± 54	32.1 (8.7, 55.5)	**0.008**
Protein intake (g/day)	142 ± 46	128 ± 41	17.6 (1.5, 33.7)	**0.034**
Total energy intake (kcal/day)	4,401 ± 1,363	3,740 ± 1,034	676 (190, 1,162)	**0.007**
Total energy intake (% WMEN)	157 ± 45	133 ± 34	30.9 (4.2, 57.6)	**0.02**

SNP rs11872292 had a low allele frequency for the A allele (mAF = 0.07), therefore, the heterozygous GA and homozygous AA genotypes were combined for analyses. *p* value for percentage of body fat was adjusted for age, sex and heritage. *p* value for total energy intake was adjusted for age, sex, heritage, fat mass and fat-free mass, and *p* value for 24-h energy expenditure was additionally adjusted for spontaneous physical activity. All remaining *p* values were adjusted for age, sex, percentage of body fat and heritage. Bold values indicate *p* ≤ 0.05. Data are given as unadjusted mean. Estimate (*β*) represents the effect per copy of the risk allele after adjusting for covariates.  % WMEN is calculated as daily energy intake/WMEN ×100

To test whether rs11872992 had an effect on eating behavior, data were analyzed in 178 adult participants who had a measure of ad libitum food intake assessed over 3 days and presented as the daily average. SNP rs11872992 was associated with total energy intake, where individuals with the risk allele G for BMI had a mean increase of total energy intake by 676 (190, 1,162) kcal/day compared with the non-risk allele A carriers (Table 2, *p* = 0.007, adjusted for age, sex, fat mass, fat-free mass and heritage). When energy intake was analyzed as the percentage of weight maintaining energy needs (WMEN), the G allele was associated with an increase of 30.9 (4.2, 57.6) % WMEN (adjusted *p* = 0.02). This was reflected in a higher intake of carbohydrate [Table 2, *β* = 81.5 (21.4, 141.6) g/day, adjusted *p* = 0.007], fat [*β* = 32.1 (8.7, 55.5) g/day, adjusted *p* = 0.008] and protein [*β* = 17.6 (1.5, 33.7) g/day, adjusted *p* = 0.03] in carriers of the G allele. This finding was consistent with data obtained from a separate ad libitum food intake study conducted in 55 full-heritage Pima Indian children. Those with the risk allele for rs11872992 have a mean increase of energy intake by 504 (95, 913) kcal/day, or 19.2 (3.6, 34.7) % WMEN (both *p* = 0.02, adjusted for age, sex, BMI, mother’s energy intake and in utero exposure to T2D). However, given the small sample size or original design of the study in children, these differences should be interpreted with caution.

### Additional association analyses for all other tag SNPs

None of the individual SNPs, with the exception of rs11872992 (shown in Table 2), nor the haplotype of rs74861148 and rs483145 was associated with percentage of body fat, energy expenditure, ad libitum food intake or T2D in this study (data not shown).

## Discussion

We previously identified rare functional coding variants that cause severe early-onset monogenic forms of obesity in American Indians. However, the relative contribution of common variation in/near *MC4R* to risk of obesity in this population was largely unknown. In the present study, we showed that common variation in/near *MC4R* has a modest effect on BMI risk in both children and adults. Although none of the common variants achieved genome-wide significance (*p* < 7.2 × 10^−8^, Dudbridge and Gusnanto 2008), the role of MC4R in the development of obesity is well established, thus applying such stringent criteria for statistical significance in this study may be overly conservative. Conditional analyses identified that rs74861148 and rs483125 independently contributed to BMI risk, and an additive effect of these two SNPs on BMI was evident. Individuals with four risk alleles (13 % of the population) had an adjusted BMI that was 3.3 kg/m^2^ greater than those with 0 or 1 risk allele (19 % of the population). In children, the adjusted Δ*z* score between these two groups was 0.23, corresponding to a ΔBMI of 1.4 kg/m^2^. In comparison, children with or without rare coding functional *MC4R* mutations had a Δ*z* score = 0.89 (corresponding to ΔBMI = 4.8 kg/m^2^) (Thearle et al. 2012). Although the effect size of common variation in *MC4R* is smaller than that of the rare coding variants, because of the greater frequency/prevalence within the population, the common SNPs are likely to have a greater overall impact on obesity predisposition at the population level.

Common variants near *MC4R* have been reproducibly associated with BMI in GWAS of Caucasians and other populations (Loos et al. 2008; Willer et al. 2009; Speliotes et al. 2010). Several widely replicated variants in Caucasians: rs17782313, rs17700633 and rs571312 were of low frequency or rare in American Indians (mAF = 0.03, 0.005 and 0.003, respectively), hence the statistical power was limited in this study; whereas the strongest BMI signal rs74861148 in American Indians (mAF = 0.38) was monomorphic in Caucasians and Africans and had a mAF of 0.03 in Asians, indicating that American Indians may harbor a population specific BMI signal in/near *MC4R*. A functional promoter SNP rs11872992 had a limited statistical power due to a low minor allele frequency in Pima Indians (mAF = 0.07), thus only achieved a nominal associations with BMI (*p* = 0.05 in adult BMI; *p* = 0.01 in childhood *z* score), despite a considerable/comparable effect size (*β* = 0.61 kg/m^2^ in adult BMI; 0.11 in childhood *z* score). This SNP was also associated with obesity in a case–control study of the Belgian population (mAF = 0.11, *p* = 0.03) (Beckers et al. 2011), but not associated with BMI in the GIANT study (mAF = 0.13, *p* = 0.19), nor in a case–control study (246 obese/482 lean subjects) of the Finnish population (mAF = 0.13, *p* = 0.57) (Valli-Jaakola et al. 2008). Therefore, future replications are required to further validate the significance of this SNP.

MC4R haploinsufficiency is associated with hyperphagia, binge eating, decreased energy expenditure and obesity (Farooqi et al. 2003; Branson et al. 2003). However, reports of the association of common variants in/near *MC4R* with energy intake are scarce and controversial. The established variant rs17782313 near *MC4R* was associated with increased snacking behavior and a higher hunger score on the Three-Factor Eating Questionnaire (TFEQ) (Stutzmann et al. 2009; Stunkard and Messick 1985). An association with higher intake of total energy and dietary fat was reported (Qi et al. 2008), but could not be confirmed (Hasselbalch et al. 2010). In these studies, eating behavior traits were all assessed through self-reported questionnaires. Our study is the first to report a positive association of a common variant rs11872992 with daily food intake using an automated vending machine paradigm in a clinical research unit. At rs11872992, the BMI risk allele carriers had a substantial increase of 676 kcal/day or 31 % WMEN in total energy intake. A separate study in children provided confirmatory support. However, it is unknown why the haplotype G–A of rs74861148 and rs483145, which had the strongest association with BMI in this study (*p* = 5.5 × 10^−7^) was not associated with increased food intake. Although ad libitum food intake in this setting was higher than the WMEN, this system provides a more reproducible (intra-class correlation coefficient 0.9) and accurate measure of food intake than that afforded by methods based on self-report (Venti et al. 2010; Tarasuk and Beaton 1992a, b). In addition to increased food intake, the risk allele at rs11872992 was also associated with a small reduction in energy expenditure (by 53 kcal/day) which was consistent with, although less than that observed in a prior report with the rare coding mutations in Pima Indians (by 110–140 kcal/day) (Krakoff et al. 2008).

Horstmann et al. (2013) reported that rs17782313 near *MC4R* has a gender-specific impact (women only) on human brain structure and eating behavior. Consistent with this observation of a gender-specific effect near *MC4R*, one SNP in our study (rs62097832 in Table 1) had a gender-specific interaction (*p* = 0.03), and was associated with maximum BMI in women alone (*p* = 1 × 10^−5^), but not in men alone (*p* = 0.38).

SNP rs11872992 had a modest functional effect in vitro. However, we have not systematically conducted functional analyses of rs74861148 or rs483145 or variants tagged by rs483145. The ENCODE database (http://www.broadinstitute.org/mammals/haploreg) predicts that rs74861148 may alter a regulatory motif for Hoxa5 binding, while rs483145 could potentially affect several motifs including FOXA1, HNF4A and Pbx1, etc., implicating a role in transcriptional regulation. While rs74861148 is a singleton, rs483145 tags 31 variants >55 kb upstream of *MC4R* in Pima Indians, many of which map to transcription factor binding motifs, and two (rs10871778 and rs11661934) map to DNAse hypersensitive sites (ENCODE). If any of these variants are functional, it could explain the stronger associations with BMI observed in American Indians.

In conclusion, in this population-based study, both rare and common variants in/near *MC4R* influence obesity in American Indian children and adults. We have identified a common haplotype G–A of rs74861148 and rs483145, which provides evidence for association of *MC4R* with increased BMI risk in American Indians. Our longitudinal data with repeated BMI measures allowed a comprehensive assessment for the effect of MC4R on obesity risk over lifetime (age 5–50 years). Moreover, a promoter variant rs11872992 in *MC4R* influences risk of obesity, perhaps in part through a propensity for increased food intake and decreased energy expenditure. Our findings provide an example of the overlap in genetic determinants of monogenic and complex obesity.

## Electronic supplementary material

Below is the link to the electronic supplementary material.

Supplemental Table. Association results for tag SNPs in the *MC4R* region with maximum BMI during adulthood and maximum BMI *z* score during childhood in full-heritage Pima Indians (n = 56 tag SNPs) and mixed-heritage American Indians (n = 8 tag SNPs). For adult BMI analyses, BMI was log_e_ transformed, and the regression coefficient (Beta) was exponentiated to obtain the effect estimate for each risk allele, expressed as a multiplier. For presentation in the table, a multiplier was converted to the effect size in kg/m^2^ based on a percentage of risk increase or decrease in mean population BMI (35.12 kg/m^2^). Beta for child BMI was expressed as *z* score per copy of the risk allele. Beta and *p* values were adjusted for age, sex, birth year and heritage. Supplemental Figure. Relative positions and pair-wise linkage disequilibrium (LD) plot for 56 common tag SNPs across the *MC4R* region (chr18:57778138-58288450, GRCh37/hg19) in full-heritage Pima Indians. LD is shown as r^2^, and tag SNP is determined by r^2^ ≥ 0.8 taken as indicative of redundancy. (DOCX 415 kb)
