# Supplementary material for: Common genetic variation in and near the melanocortin 4 receptor gene (MC4R) is associated with body mass index in American Indian adults and children
Source: Hum Genet. 2014 Aug 8;133(11):1431–41. doi: 10.1007/s00439-014-1477-6 (PMC4185108; doi:10.1007/s00439-014-1477-6)

Supplemental Table.

|  |  |  | Full-heritage Pima Indian  Adults (n=2862), Children (=2296) | | | | | |  | Mixed-heritage American Indian  Adults (n=3056), Children (n=3054) | | | | | |
| --- | --- | --- | --- | --- | --- | --- | --- | --- | --- | --- | --- | --- | --- | --- | --- |
| SNPs | Age | Risk  /Non | RAF | Risk/  Risk | Risk/  Non | Non/  Non | Beta | P value |  | RAF | Risk/  Risk | Risk/  Non | Non/  Non | Beta | P Value |
| rs185289287 | Adult BMI  BMI z-score | G/A | 0.93 | 36.5  0.27 | 36.7  0.39 | 34.6  0.25 | 0.386  -0.106 | 0.355  0.123 |  |  |  |  |  |  |  |
| rs74861148 | Adult BMI  BMI z-score | G/A | 0.45 | 37.5  0.34 | 36.1  0.30 | 35.6  0.21 | 0.632  0.048 | **0.004**  0.120 |  | 0.32 | 35.7  0.50 | 34.4  0.32 | 33.2  0.28 | 0.562  0.054 | **0.009**  0.097 |
| rs1673518 | Adult BMI  BMI z-score | T/C | 0.95 | 36.4  0.29 | 36.0  0.24 | 33.2  -0.90 | 0.316  0.025 | 0.521  0.725 |  |  |  |  |  |  |  |
| rs17782271 | Adult BMI  BMI z-score | A/C | 0.85 | 36.5  0.28 | 35.6  0.30 | 36.6  0.25 | 0.457  0.001 | 0.119  0.993 |  |  |  |  |  |  |  |
| rs567792 | Adult BMI  BMI z-score | G/C | 0.96 | 36.5  0.29 | 35.6  0.21 | 30.3  1.06 | 0.246  -0.015 | 0.694  0.873 |  |  |  |  |  |  |  |
| rs11152214 | Adult BMI  BMI z-score | G/T | 0.80 | 36.6  0.30 | 36.2  0.27 | 36.0  0.11 | 0.351  0.050 | 0.210  0.214 |  |  |  |  |  |  |  |
| rs9319962 | Adult BMI  BMI z-score | T/G | 0.83 | 36.4  0.29 | 36.1  0.29 | 36.1  0.30 | 0.351  0.025 | 0.272  0.582 |  |  |  |  |  |  |  |
| rs77262357 | Adult BMI  BMI z-score | C/T | 0.96 | 36.5  0.28 | 35.9  0.29 | 35.9  -0.07 | 0.632  0.103 | 0.263  0.196 |  |  |  |  |  |  |  |
| rs143262006 | Adult BMI  BMI z-score | T/C | 0.87 | 36.6  0.29 | 35.8  0.26 | 35.8  -0.07 | 0.492  0.051 | 0.127  0.290 |  |  |  |  |  |  |  |
| rs35623600 | Adult BMI  BMI z-score | A/C | 0.90 | 36.4  0.29 | 36.1  0.25 | 36.3  0.34 | 0.246  0.054 | 0.495  0.291 |  |  |  |  |  |  |  |
| chr18:57983977 | Adult BMI  BMI z-score | T/A | 0.96 | 36.5  0.29 | 35.8  0.18 | 36.4  0.79 | 0.738  0.100 | 1.130  0.199 |  |  |  |  |  |  |  |
| rs142153873 | Adult BMI  BMI z-score | G/A | 0.95 | 36.4  0.27 | 35.6  0.40 | 36.1  0.78 | 0.316  -0.129 | 0.532  0.080 |  |  |  |  |  |  |  |
| rs137860068 | Adult BMI  BMI z-score | G/A | 0.95 | 36.5  0.28 | 36.0  0.31 | 33.3  -0.34 | 0.773  0.088 | 0.107  0.232 |  |  |  |  |  |  |  |
| rs1943214 | Adult BMI  BMI z-score | C/T | 0.61 | 36.8  0.32 | 36.4  0.31 | 35.6  0.15 | 0.457  0.040 | **0.035**  0.221 |  |  |  |  |  |  |  |
| rs8087522 | Adult BMI  BMI z-score | A/G | 0.49 | 36.8  0.35 | 36.3  0.32 | 35.4  0.18 | 0.562  0.084 | **0.010**  **0.008** |  | 0.42 | 34.5  0.44 | 34.1  0.32 | 33.0  0.27 | 0.492  0.065 | **0.023**  **0.035** |
| rs11872992 | Adult BMI  BMI z-score | G/A | 0.94 | 36.2  0.32 | 35.9  0.17 | 34.7  0.07 | 0.316  0.068 | 0.513  0.296 |  | 0.91 | 34.1  0.35 | 33.3  0.19 | 31.0  -0.02 | 0.667  0.115 | 0.088  **0.029** |
| rs190062199 | Adult BMI  BMI z-score | A/G | 0.02 |  | 36.3  0.56 | 36.2  0.28 | 0.386  0.230 | 0.640  0.122 |  |  |  |  |  |  |  |
| rs62097830 | Adult BMI  BMI z-score | A/C | 0.91 | 36.5  0.29 | 36.1  0.23 | 36.0  0.20 | 0.457  0.090 | 0.210  0.083 |  |  |  |  |  |  |  |
| rs62097832 | Adult BMI  BMI z-score | G/A | 0.59 | 36.8  0.36 | 36.3  0.28 | 35.1  0.14 | 0.667  0.094 | **0.002**  **0.001** |  | 0.47 | 35.2  0.49 | 34.0  0.29 | 33.2  0.24 | 0.457  0.068 | **0.046**  **0.038** |
| rs6567167 | Adult BMI  BMI z-score | C/T | 0.73 | 36.5  0.31 | 36.3  0.28 | 35.2  0.12 | 0.421  0.042 | 0.093  0.225 |  |  |  |  |  |  |  |
| rs11661166 | Adult BMI  BMI z-score | G/A | 0.60 | 36.8  0.39 | 36.0  0.26 | 35.6  0.13 | 0.492  0.092 | **0.026**  **0.004** |  | 0.52 | 34.6  0.42 | 34.0  0.31 | 33.3  0.25 | 0.421  0.070 | 0.063  **0.023** |
| rs34092067 | Adult BMI  BMI z-score | A/G | 0.46 | 37.0  0.36 | 36.3  0.30 | 36.0  0.17 | 0.457  0.077 | **0.031**  **0.012** |  |  |  |  |  |  |  |
| rs9966038 | Adult BMI  BMI z-score | G/C | 0.78 | 36.6  0.32 | 36.2  0.22 | 36.0  0.21 | 0.281  0.047 | 0.282  0.212 |  |  |  |  |  |  |  |
| rs11152222 | Adult BMI  BMI z-score | G/T | 0.58 | 36.4  0.29 | 36.5  0.33 | 35.5  0.18 | 0.351  0.025 | 0.092  0.425 |  |  |  |  |  |  |  |
| rs8088123 | Adult BMI  BMI z-score | C/A | 0.86 | 36.3  0.33 | 35.8  0.17 | 36.2  0.01 | 0.105  0.111 | 0.722  **0.006** |  | 0.90 | 34.1  0.34 | 33.4  0.23 | 34.7  0.80 | 0.702  0.062 | 0.055  0.210 |
| rs11873709 | Adult BMI  BMI z-score | G/A | 0.50 | 36.3  0.29 | 36.4  0.33 | 35.7  0.20 | 0.316  0.038 | 0.133  0.209 |  |  |  |  |  |  |  |
| rs948810 | Adult BMI  BMI z-score | G/A | 0.16 | 36.3  0.28 | 36.7  0.34 | 36.2  0.27 | 0.140  0.052 | 0.651  0.230 |  |  |  |  |  |  |  |
| rs11152223 | Adult BMI  BMI z-score | A/G | 0.38 | 36.0  0.30 | 36.8  0.32 | 36.0  0.23 | 0.316  0.041 | 0.163  0.218 |  |  |  |  |  |  |  |
| rs1943241 | Adult BMI  BMI z-score | C/T | 0.67 | 36.2  0.22 | 36.7  0.35 | 36.3  0.25 | 0.105  -0.348 | 0.661  0.323 |  |  |  |  |  |  |  |
| rs78505826 | Adult BMI  BMI z-score | G/T | 0.10 | 38.8  0.32 | 36.6  0.25 | 36.4  0.29 | 0.140  -0.049 | 0.723  0.340 |  |  |  |  |  |  |  |
| rs2115922 | Adult BMI  BMI z-score | A/T | 0.78 | 36.5  0.31 | 35.9  0.26 | 35.1  0.10 | 0.457  0.063 | 0.092  0.083 |  |  |  |  |  |  |  |
| rs2156334 | Adult BMI  BMI z-score | G/T | 0.21 | 37.5  0.43 | 36.6  0.31 | 36.2  0.25 | 0.140  0.034 | 0.536  0.360 |  |  |  |  |  |  |  |
| chr18:58146264 | Adult BMI  BMI z-score | A/G | 0.98 | 36.4  0.29 | 36.4  0.10 |  | 0.316  0.178 | 0.694  0.086 |  |  |  |  |  |  |  |
| rs73461644 | Adult BMI  BMI z-score | C/T | 0.51 | 36.6  0.30 | 36.6  0.29 | 36.0  0.25 | 0.281  0.019 | 0.190  0.556 |  |  |  |  |  |  |  |
| rs483145 | Adult BMI  BMI z-score | A/T | 0.81 | 36.5  0.33 | 35.8  0.21 | 35.0  0.02 | 0.351  0.098 | 0.193  **0.006** |  | 0.79 | 34.6  0.36 | 33.0  0.24 | 33.0  0.35 | 0.738  0.046 | **0.003**  0.192 |
| rs138456532 | Adult BMI  BMI z-score | C/G | 0.10 | 39.5  0.38 | 36.9  0.36 | 36.3  0.26 | 0.386  0.060 | 0.303  0.268 |  |  |  |  |  |  |  |
| chr18:58169913 | Adult BMI  BMI z-score | T/G | 0.05 | 37.0  -0.09 | 37.1  0.30 | 37.4  0.28 | 0.773  -0.011 | 0.150  0.875 |  |  |  |  |  |  |  |
| rs553429 | Adult BMI  BMI z-score | T/A | 0.71 | 36.8  0.35 | 36.1  0.22 | 36.2  0.17 | 0.246  0.077 | 0.310  **0.036** |  |  |  |  |  |  |  |
| rs17067025 | Adult BMI  BMI z-score | G/A | 0.96 | 36.5  0.29 | 35.5  0.19 | 39.5  1.37 | 0.527  0.064 | 0.230  0.369 |  |  |  |  |  |  |  |
| rs2156608 | Adult BMI  BMI z-score | A/G | 0.63 | 36.7  0.36 | 36.0  0.25 | 35.7  0.17 | 0.316  0.063 | 0.142  0.047 |  |  |  |  |  |  |  |
| rs12606080 | Adult BMI  BMI z-score | A/T | 0.82 | 36.4  0.33 | 36.1  0.18 | 36.4  0.20 | 0.105  0.080 | 0.690  **0.045** |  |  |  |  |  |  |  |
| rs881475 | Adult BMI  BMI z-score | A/G | 0.15 | 38.2  0.25 | 36.3  0.28 | 36.2  0.28 | 0.211  -0.048 | 0.471  0.246 |  |  |  |  |  |  |  |
| rs648466 | Adult BMI  BMI z-score | G/A | 0.83 | 36.5  0.29 | 35.9  0.27 | 36.2  0.34 | 0.351  -0.005 | 0.240  0.912 |  |  |  |  |  |  |  |
| rs637188 | Adult BMI  BMI z-score | C/G | 0.34 | 37.1  0.20 | 36.0  0.25 | 36.4  0.34 | 0.105  -0.073 | 0.694  **0.028** |  |  |  |  |  |  |  |
| rs474421 | Adult BMI  BMI z-score | C/T | 0.55 | 36.7  0.39 | 35.8  0.26 | 36.4  0.21 | 0.211  0.086 | 0.323  **0.007** |  | 0.54 | 34.0  0.27 | 34.2  0.36 | 33.6  0.30 | 0.105  -0.017 | 0.684  0.563 |
| rs530676 | Adult BMI  BMI z-score | T/G | 0.73 | 36.4  0.30 | 36.0  0.29 | 36.6  0.21 | 0.211  0.056 | 0.368  0.102 |  |  |  |  |  |  |  |
| rs695020 | Adult BMI  BMI z-score | C/A | 0.89 | 36.3  0.30 | 35.8  0.24 | 35.2  0.25 | 0.492  0.040 | 0.127  0.399 |  |  |  |  |  |  |  |
| rs4940946 | Adult BMI  BMI z-score | C/T | 0.32 | 36.7  0.29 | 36.1  0.28 | 36.5  0.29 | 0.000  -0.024 | 0.957  0.488 |  |  |  |  |  |  |  |
| rs551347 | Adult BMI  BMI z-score | A/G | 0.05 | 33.8  0.26 | 36.4  0.38 | 36.5  0.27 | 0.316  0.097 | 0.502  0.185 |  |  |  |  |  |  |  |
| rs183432256 | Adult BMI  BMI z-score | A/G | 0.96 | 36.4  0.29 | 36.4  0.24 | 36.8  -0.99 | 0.246  0.109 | 0.677  0.142 |  |  |  |  |  |  |  |
| rs1991613 | Adult BMI  BMI z-score | C/G | 0.41 | 36.9  0.35 | 36.4  0.30 | 36.4  0.24 | 0.140  0.059 | 0.538  0.070 |  |  |  |  |  |  |  |
| rs512279 | Adult BMI  BMI z-score | A/G | 0.21 | 35.9  0.13 | 36.4  0.26 | 36.5  0.30 | 0.176  -0.040 | 0.459  0.279 |  |  |  |  |  |  |  |
| rs8088802 | Adult BMI  BMI z-score | G/A | 0.69 | 36.5  0.34 | 35.9  0.25 | 36.1  0.19 | 0.140  0.073 | 0.592  **0.022** |  |  |  |  |  |  |  |
| rs603119 | Adult BMI  BMI z-score | A/G | 0.23 | 37.3  0.24 | 36.4  0.30 | 36.4  0.29 | 0.140  -0.026 | 0.611  0.506 |  |  |  |  |  |  |  |
| rs5020834 | Adult BMI  BMI z-score | T/C | 0.54 | 36.6  0.25 | 36.3  0.27 | 36.6  0.37 | 0.105  -0.054 | 0.578  0.100 |  |  |  |  |  |  |  |
| rs9952630 | Adult BMI  BMI z-score | G/A | 0.11 | 39.1  0.55 | 36.4  0.30 | 36.3  0.27 | 0.316  0.053 | 0.369  0.332 |  |  |  |  |  |  |  |


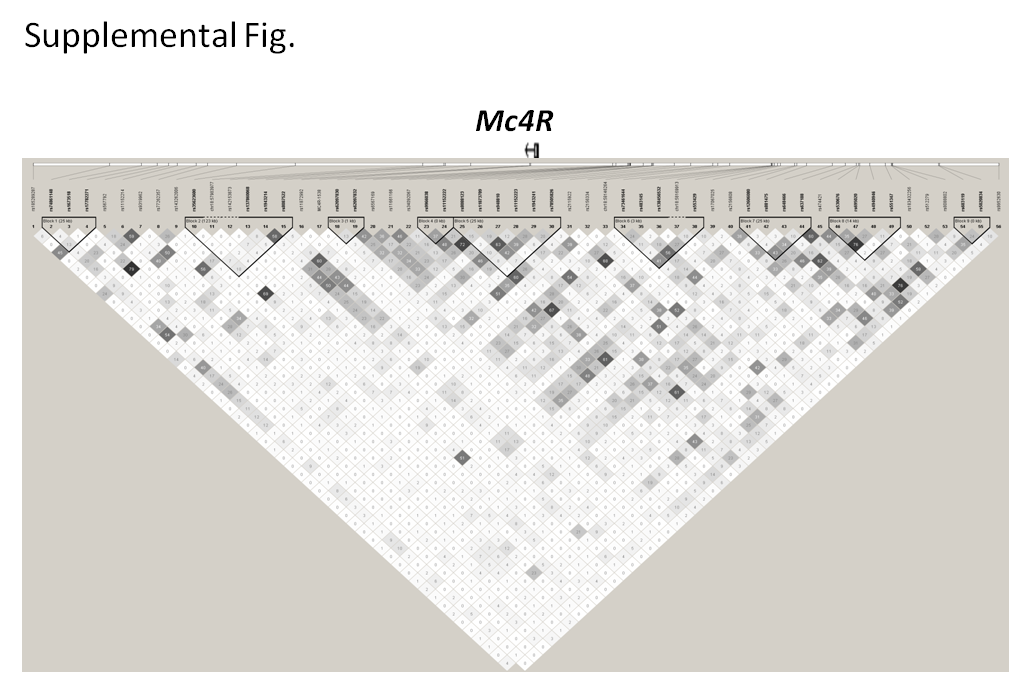

Supplement: Supplementary file 1 — Supplemental Table. Association results for tag SNPs in the MC4R region with maximum BMI during adulthood and maximum BMI z score during childhood in full-heritage Pima Indians (n = 56 tag SNPs) and mixed-heritage American Indians (n = 8 tag SNPs). For adult BMI analyses, BMI was loge transformed, and the regression coefficient (Beta) was exponentiated to obtain the effect estimate for each risk allele, expressed as a multiplier. For presentation in the table, a multiplier was converted to the effect size in kg/m2 based on a percentage of risk increase or decrease in mean population BMI (35.12 kg/m2). Beta for child BMI was expressed as z score per copy of the risk allele. Beta and p values were adjusted for age, sex, birth year and heritage. Supplemental Figure. Relative positions and pair-wise linkage disequilibrium (LD) plot for 56 common tag SNPs across the MC4R region (chr18:57778138-58288450, GRCh37/hg19) in full-heritage Pima Indians. LD is shown as r2, and tag SNP is determined by r2 ≥ 0.8 taken as indicative of redundancy. (DOCX 415 kb) [file 439_2014_1477_MOESM1_ESM.docx]
